# Synthesis, Optical Characterizations and Solar Energy Applications of New Schiff Base Materials

**DOI:** 10.3390/ma14133718

**Published:** 2021-07-02

**Authors:** Sobhi M. Gomha, Hoda A. Ahmed, Mohamed Shaban, Tariq Z. Abolibda, Muna S. Khushaim, Khalid A. Alharbi

**Affiliations:** 1 Chemistry Department, Faculty of Science, Islamic University in Almadinah Almonawara, Almadinah Almonawara 42351, Saudi Arabia; t.z.a@iu.edu.sa (T.Z.A.); k.abdulazizalharbi@gmail.com (K.A.A.); 2 Department of Chemistry, Faculty of Science, Cairo University, Cairo 12613, Egypt; 3 Chemistry Department, College of Sciences, Yanbu, Taibah University, Yanbu 30799, Saudi Arabia; 4 Department of Physics, Faculty of Science, Islamic University in Almadinah Almonawara, Almadinah 42351, Saudi Arabia; mssfadel@aucegypt.edu; 5 Department of Physics, Faculty of Science, Taibah University, P.O. Box 30002, Al-Madina 41447, Saudi Arabia; mkhushaim@taibahu.edu.sa; 6 Nanotechonolgy Center, Taibah University, P.O. Box 30002, Al-Madina 41447, Saudi Arabia

**Keywords:** lateral methoxy schiff base/ester, optical properties, mesophase stability, photophysical, solar energy, electrical properties

## Abstract

A new set of laterally OCH_3_-substituted photoactive liquid crystalline analogues, 4-hexyloxy phenyl- imino-4ʹ-(3-methoxyphenyl)-4ʹ’-alkoxybenzoates, were synthesized and investigated for their mesomorphic behavior. The prepared set constitutes five analogues that differ from each other by the terminally attached compact polar group. Characterization of the synthesized derivatives is conducted using differential scanning calorimetry (DSC), polarized optical microscopy (POM), and UV-spectroscopy. Molecular structures were elucidated by elemental analyses, FT-IR and NMR spectroscopy. DSC and POM investigations indicated that all the prepared derivatives are monomorphic possessing the nematic (N) phase, except for the unsubstituted derivative that is nonmesomorphic. On the other side, the photophysical study and the optical spectra measurements confirm the photoactivity of the present compounds under UV/visible irradiation. The measured optical spectra showed impressive enhancement in the optical absorption and reduction in the optical bandgap from 3.63 to 3.0 eV depending on the terminal group. From the study of the DC electric properties, the lowest resistance, 106.5 GΩ at scan rate 0.1 V/s, was observed for the **I6_d_** film with Cl terminal, which decreased to 49.5 GΩ by increasing the scan rate to 0.5 V/s. Moreover, the electrical conductance is decreased from 9.39 pS to 1.35 pS at scan rate 0.1 V/s by changing the terminal group from Cl to F. The enhanced optical absorption and the reduced energy gap make the optimized samples suitable material for solar energy applications.

## 1. Introduction

Nowadays, liquid crystalline (LC) compounds prove to have several applications as optical materials—areas of technological applications such as displays, light-emitting diodes based on organic derivatives, anisotropic networks, and semi- and photoconductors materials [1,2,3]. The most helpful characteristics of these LC compounds that are considered to be essential in applications in devices are the mesophase type, switching times and optical rotation-power near the working temperatures [4]. Geometrical–property relationships could be important tools to synthesize suitable architectures to achieve the desired properties for device-displays [5,6,7,8]. Consequently, the selection of the flexible alkoxy/alkyl groups, terminal wings, as well as mesogenic linkers, is an important criterion in the formation of thermotropic LCs for proper characteristic industries. Moreover, the molecular shape enables some essential changes in the mesomorphic characteristics and plays an important role in the formation, kind, and thermal stability of the formed mesophase [9,10,11,12,13,14,15,16].

On the other hand, metal-containing LCs (metallomesogens) have received considerable attention because of their possibility to combine the characteristics of transition metals with the self-organization of the liquid crystalline mesophase [17,18]. Moreover, the mobility of the photogenerated charges is greatly enhanced due to the unidirectional transport in the disk-shaped LC structural materials. Discotics are considered as an essential class of organic semiconductors [19]. Moreover, extensive researches have also been focused on the field of LC polymers due to their importance in the medical and industrial fields [20,21]. Generally, the insertion of lateral-substituent increases the intermolecular separations, which widen the core moiety that leads to a decrement in the lateral interactions [22,23,24]. In addition, as the breadth of the molecule increases, the thermal stability of both smectic and nematic phases are reduced [25]. The small volume of the lateral group enables its additions into the mesomorphic architectures without being sterically disrupted, and consequently, its LC mesophases can still be observed. It has also been documented that lateral or terminal polar substituent induces an effect on the mesomeric properties of huge numbers of Schiff bases/ester derivatives [26,27,28,29].

Solar energy has the potential to be one of the most important sources by 2050. With such solar photovoltaic and concentrated solar energy, it will contribute more than 16% and 11% of the total world energy needs [30]. Organic solar cells are known as favorable cheap renewable energy technology [31,32,33]. Organic thin-film solar cells are promising types for flat-plate terrestrial applications because of their possibly low-cost methods. For solar energy investigtions such as solar-hydrogen processing, photoelectrochemical water splitting, solar cells, and photocatalytic dye degradation; the band gap engineering and optical property control are very relevant [34,35,36,37,38].

Previously, based on Schiff base/ester central linkages, sets of terminal substituted derivatives were synthesized and investigated for their mesomorphic properties; in addition, the effect of the terminal long and compact group polarities on the mesophase were also studied [9,10,11,12,13,14,15,16]. On this basis, the goal of the present study is to synthesize new laterally methoxy Schiff base derivatives, with different terminal polar compact groups (X), namely, 4-(((4-(hexyloxy)phenyl)imino)methyl)-3-methoxyphenyl 4-substituted benzoates **I6_a–e_**.

**Figure 1:**
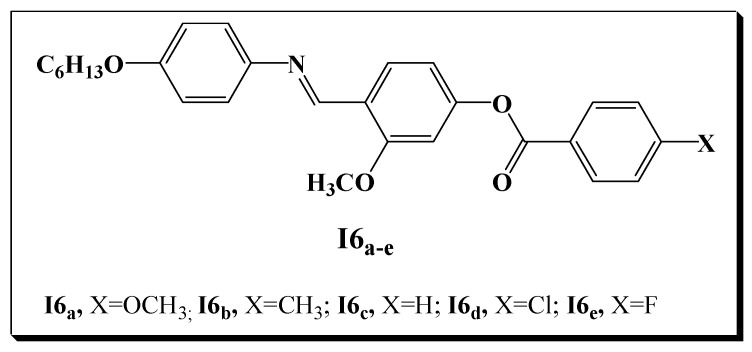
Prepared homologues series, **I6_a–e_**.

On the phenyl azomethine group, hexadecyloxy group is attached, while on the other terminal phenyl ring, different sized polar substituents (OCH_3_, CH_3_, H, Cl, F) are attached, and a lateral methoxy group is attached to the central ring (Figure 1). Further aim of the study is to investigate the mesophase and photo-physical behaviors as well as the effect of the change of the polar terminal substituents on the mesomorphic properties of synthesized compounds. Moreover, the electrical as well as the optical band gap energy and band tail are calculated as a function of the terminal polar group. Finally, a comparison is conducted between the present laterally substituted series and the previously investigated laterally neat analogues in order to investigate the effect of the protrusion of the lateral OCH_3_ group on the mesomorphic characters.

## 2. Experimental

### 2.1. Synthesis

**Scheme 1:**
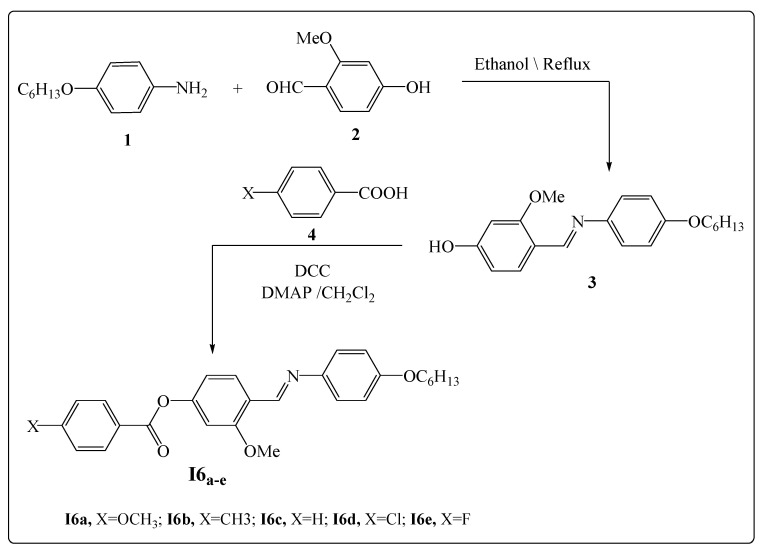
Synthesis route of title compounds **I6_a–e_**.

The synthesis pathway leading to the title compounds **I6_a–e_** are presented in Scheme 1 and details of preparations with spectroscopic analyses were inserted in Supplementary Data.

### 2.2. Films Preparation

A very thin layer of the sample was prepared by sandwiching them between a glass slide and a coverslip. The dimensions of the cell were 22 mm × 22 mm × 0.03 mm, i.e., the film thickness was ~30 μm. The temperature of the cell was controlled using a temperature controller with an accuracy ±0.1 °C.

## 3. Results and Discussion

### 3.1. Molecular Confirmation of Synthesized Compounds **I6_a–e_**

Schiff base and hydrazone derivatives are well known as valuable intermediates in synthesis of many organic compounds that exhibit many applications [39,40,41,42,43,44,45,46]. A series of new laterally methoxy Schiff base derivatives, with different terminal polar compact groups (X), namely, 4-(((4-(hexyloxy)phenyl)imino)methyl)-3-methoxyphenyl 4-substituted benzoates **I6_a–e_**, have been prepared as shown in Scheme 1.

Molecular structures of products **I6_a–e_** were elucidated via their spectroscopic data αFT IR, ^1^H-NMR and ^13^C-NMR, see Supplementary Data) and elemental analyses. 

The FT-IR spectra of compounds **I6_a–e_** revealed in each case two characteristic absorption bands in the regions υ 1720–1731, 1606–1612 cm^−1^ attributed to the C=O and C=N groups. The FT-IR results revealed that compounds **I6_d_** and **I6_e_** (X=Cl or F, electron withdrawing groups) had no pronounced effect on the absorption band of C=O group compared to the unsubstituted derivative **I6_c_** (X=H; *v* = 1723, X=Cl; *v* = 1725 and X=F; *v* = 1722 cm^−1^), while compounds **I6_a_** and **I6_b_** (X=OCH_3_ or CH_3_, electron donating groups) showed marked increase in carbonyl absorption bands to 1731 and 1728 cm^−1^, respectively). In addition, the polarity of X has a slight effects on the absorption band of C=N group. The wave number of the C=N group for the unsubstituted derivative **I6_c_** appeared at 1612 cm^−1^ while the electron donating derivatives (X=CH_3_O or CH_3_) appeared near 1608 cm^−1^ instead of 1606 cm^−1^ for the electron withdrawing groups (X=F or Cl). Thus, the substituent X, regards of electron donating or electron withdrawing did not mesomerically affect the imino group.

### 3.2. Mesomorphic and Optical Properties

**Figure 2:**
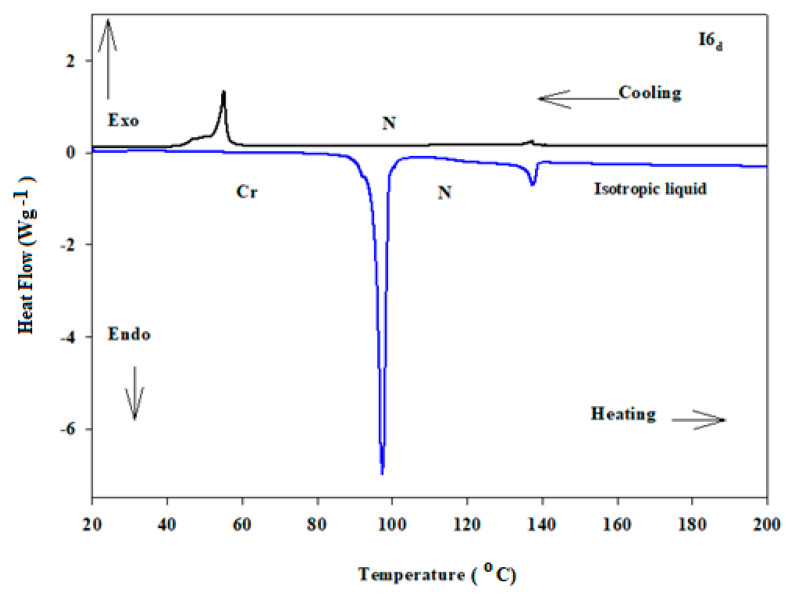
DSC thermograms of compounds **I6_d_** at a rate of ±10 °C min^−1^ recorded from the second heating and cooling cycles.

**Figure 3:**
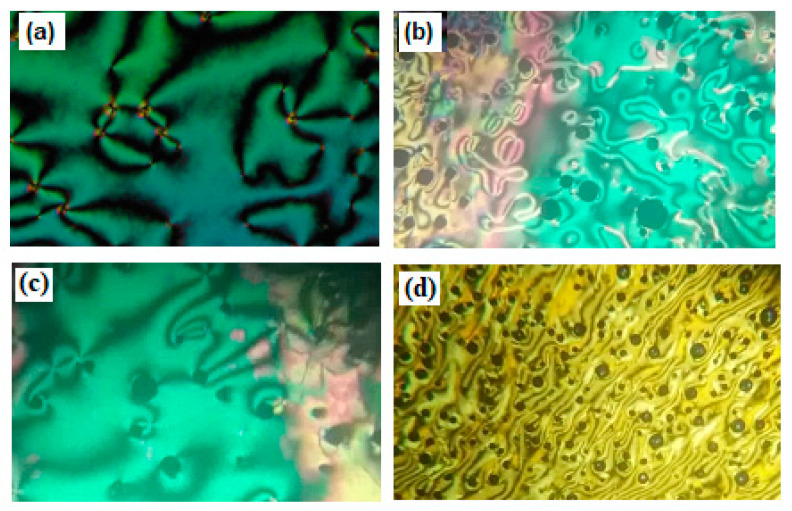
Nematic phase textures observed under POM for (**a**) compound **I6_a_** at 131.0 °C upon heating; (**b**) compound **I6_d_** at 125.0 °C upon heating; (**c**) compound **I6_d_** at 100.0 °C upon cooling; (**b**) compound **I6_a_** at 102.0 °C upon cooling.

The mesophase properties of all the synthesized derivatives have been evaluated by DSC and POM. Typical heating/cooling DSC thermograms of prepared compound **I6_d_** are displayed in Figure 2. Upon heating scans, significant endothermic peaks were observed, dependent on the polarity of terminal group (X), which are ascribed to mesomorphic transition, and the cooling cycle confirmed those observed upon decreasing the temperature. DSC curves confirm the mesophase transitions from Cr→ N, and N→ I according to the structural shape of synthesized compounds, **I6_a–e_**. POM optical images of some derivatives are illustrated in Figure 3 as examples. The nematic mesophase was identified by schlieren/threads textures observations upon heating and cooling cycle.

**Figure 4:**
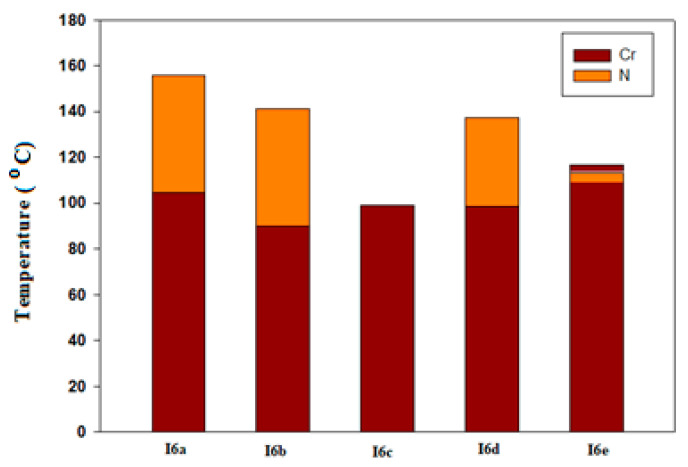
Impact of terminal polar groups on the mesomorphic behavior of the present analogous series, **I6_a–e_**.

The mesophase transition temperatures, as measured from DSC thermal analysis and their associated enthalpies for all the synthesized compounds, **I6_a–e_**, are tabulated in Table 1, and the phases transitions are depicted in Figure 4. As can be seen from Table 1 and Figure 4, all prepared compounds are mesomorphic in nature with mesomorphic range of stability dependent on their terminal substituent, except the unsubstituted derivative (**I6_c_**, X=H) which is nonmesomorphic. Moreover, all the compounds are monomorphic, possessing only the nematic phase. The mesomorphic range and stability increased in this order:

CH_3_O > CH_3_ > Cl > F > H

It can also be seen from Table 1 and Figure 4 that the melting temperature varies irregularly. Compound **I6_a_** (X=OCH_3_) has enantiotropic nematic phase with the highest nematic stability and temperature range 155.9 and 51.4 °C, respectively. The second derivative bearing the electron-donating group (**I6_b_**, X=CH_3_) also possesses enantiotropic nematic mesophase with stability and temperature range nearly 141.3 and 51.1 °C, respectively, while the derivatives bearing the electron-withdrawing groups (X=Cl and F) possess less thermal stability. Compound **I6_d_** (X=Cl) exhibits enantiotropic N phase with thermal stability 137.4 °C and range 38.7 °C. For terminal fluorine atom (**I6_e_**), it exhibits monotropic N phase with lower range of thermal stability. In general, the molecular architecture and polarizability as well as the dipole moment of the synthesized materials are highly affected by the electronic nature of the terminals and their volumes. In case of unsubstituted derivative (**I6_c_**), transitions are directly from solid to isotropic liquid upon heating and the reverse on cooling, meaning that it is nonmesomorphic. The mesomorphic stability is enhanced by an increment in the polarity and/or polarizability of the mesogenic part of the molecule. Thus, the nature of the terminal moieties (X=OCH_3_, CH_3,_ Cl and F) influences the mesophase stability and induces liquid crystalline mesophases when compared to the unsubstituted derivative (X=H). In addition, the mesophase behavior of calamitic molecules is a direct impact of molecular–molecular interactions that depend mainly on geometrical structure and space filling of the molecules, polarizability anisotropy of the polar terminal and lateral groups, the stereo electronic characters of the whole molecular shape. These factors contribute in varies extents to the mesomorphic properties observations.

The normalized entropy changes of transition, Δ*S*_N-I_/R, of the present lateral methoxy compounds (**I6_a–e_**) are collected in Table 1. Results showed that small values of the entropy changes are observed that depends on the kind of terminal substituent X. The small values observed for the entropy change can be attributed to the decrease of the length-to-breadth ratio resulting from their lower anisotropy in terms of their molecular geometry and the increment in their molecular biaxiality [47,48]. The induction, conjugation forces, the specific dipolar interactions as well as the π-π stacking interactions [49,50] play important roles in the molecular orientation and thus in the arrangement of molecules and formation of the mesophase.

### 3.3. Clearing Temperature and Polarizability Anisotropy of the C_ar_-X Bonds

The relationship between the polarizability anisotropy (Δα_X_) of bonds to the terminal compact polar group (C_ar_-X) and the thermal stability of the mesophase (clearing temperature, ***T***_C_) was investigated by van der Veen [51].

The form of relation (Equation (1)):

***T***_C_ ∝ (Δα_M_ + Δα_X_)^2^ (1)

where ***T***_C_ is measured in Kelvin. The term Δα_M_ is the anisotropy of the polarizability for whole molecular structure except the terminal group, X. Equation (1) can be put in the form (Equation(2)) [52]:

(2) $$\sqrt{T_{C}}\;\propto \;(\Delta \alpha_{M}+\Delta \alpha_{X})=a\times \;\Delta \alpha_{M}+a\times \;\Delta \alpha_{X}$$

where “*a*” is the proportionality constant. Thus, if $\sqrt{T_{C}}$ is correlated against Δα_X_ for any set of liquid crystalline materials, a straight line is expected, the slope of which equals “*a*” and intercept equals “*a*·Δα_M_”. Consequently, Δα_M_ will be given by Δα_M_ = intercept/slope.

**Figure 5:**
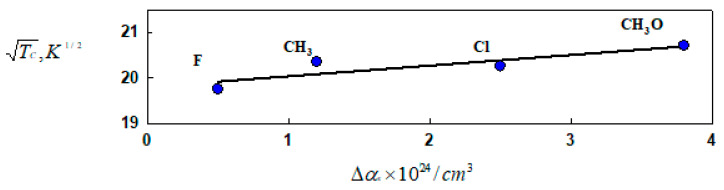
Mesophase stability ($\sqrt{T_{C}}$ ) dependency on the polarizability anisotropy (Δα_X_) of the terminal attached substituent (X) of the present series of **I6_a–e_**.

$\sqrt{T_{C}}$ values are displayed as a function of Δα_X_, for present analogous **I6_a–e_** in Figure 5. It could be seen from Figure 5, a linear correlation was obtained for all members of the synthesized series **I6_a–e_**, whereby the slope and intercept as well as Δα_M_ were calculated by the least squares method. The calculated slope and intercept have values 2.35 × 10^24^ and 19.80 K^1/2^ respectively, while the resulted Δα_M_ equal 1.18 × 10^23^ cm^3^. Additionally, a linear dependency was observed in Figure 5 for present synthesized set. Thus, the selected terminal substituents were enhancing the polarizability of the ester moiety by positive inductive effect and, consequently, the polarizability anisotropy of the whole molecular architecture.

### 3.4. Effect of the Incorporation of the Lateral Methoxy Group on the Mesomorphic Behaviors

**Figure 6:**
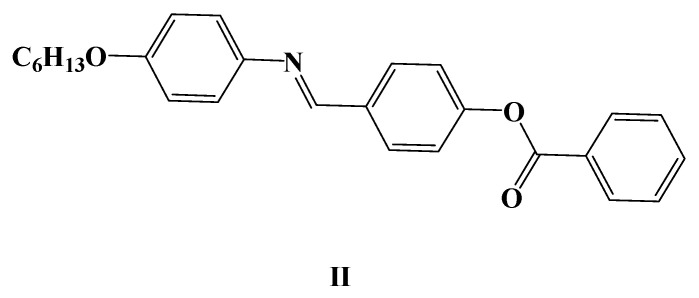
The previously laterally neat analogous, **II**.

The attachment of lateral methoxy group into the mesogenic core of the molecule will have a slight steric impact. In addition, it is an electron-donating moiety, thus it will have an affect on the intermolecular dispersion interactions. To investigate the effect of addition a lateral OCH_3_ group, in the middle ring of the molecule with special orientation, upon the mesophase and thermal behaviors of the compounds, a comparison is established between the presently prepared unsubstituted derivative (**I6_c_**) and the previously documented laterally neat analogous, **II**, Figure 6 [53] for their mesophase stabilities. Generally, the thermal stability of the formed mesophase varies according to the enhanced molecular dipole moment and polarizability of the mesogenic part, which are dependent upon the position of the lateral OCH_3_ group. Moreover, the incorporation of the lateral OCH_3_ group into the ortho position with respect to the azomethine linkage of **I6_c_** will disrupt the smectic A molecular arrangement for **II** and shows a nonmesomorphic behavior. The comparison revealed that the mesophase type and stability as well as range depends on the protrusion of the lateral OCH_3_ group which is attached within the mesogenic skeleton of the molecule.

### 3.5. Photophysical Studies

**Figure 7:**
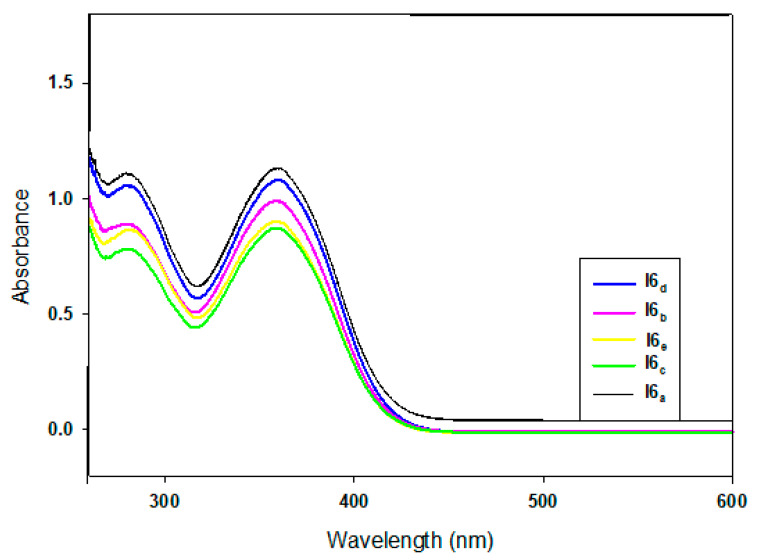
UV–vis spectrum of present synthesizes analogs series, **I6_a–e_**, in methylene chloride at 25 °C.

The reasonable thermal stabilities of liquid crystalline materials have been extensively reported for electronic and photonic applications due to their photophysical characteristics [54]. UV–vis absorption spectra were investigated for the present series **I6_a–e_**) in C = 1.0 × 10^−3^ mol L^−1^dichloromethane solution and displayed graphically in Figure 7. As can be seen from Figure 7, the radiation of light in the wavelength range 260–600 nm and the derivatives possess two strong absorption bands at ~280 nm and 360 nm, according to the dependent on the terminally attached group (X). The more intense bands observed in the analogous set, observed at a wavelength around 359–362 nm, may be assigned to the π–π* transition in the π-electronic system throughout the whole mesogenic moiety, with a suitable charge transfer (CT) property, whereas the weak absorption band that appeared around 277–280 nm may be related to the n-π* transition of the Schiff base moiety [55,56,57]. Moreover, the intensity of the peak and absorption bands is dependent on the geometrical structure of the molecule that absorbs the light at a given wavelength. Further, the present synthesized nematogenic series has promise to be photoactive organic materials for many industries fields.

### 3.6. DC Electrical Properties

**Figure 8:**
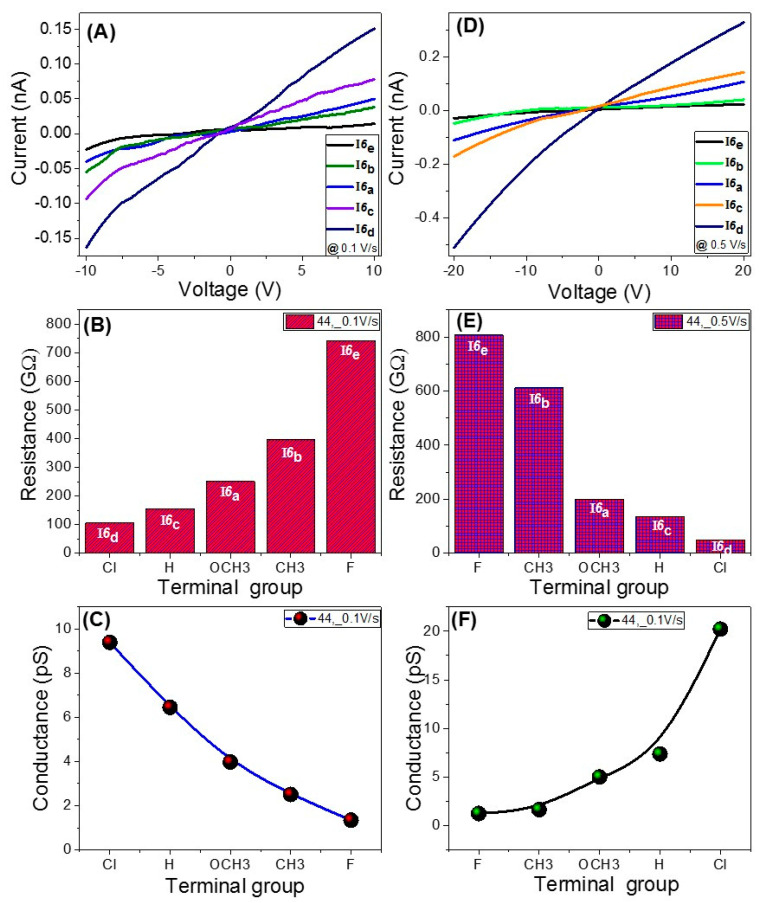
(**A**,**D**) Current-voltage characteristics, (**B**,**E**) variation of the electric resistance, and (**C**,**F**) variation of the electric conductance for the present series (**I6_a–e_**) measured at scan rates 0.1 and 0.5 V/s at 115 °C.

**Figure 9:**
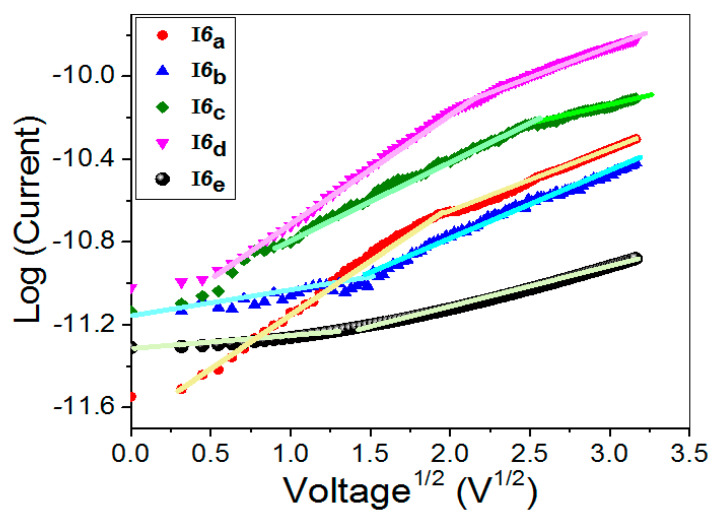
The relation between log (I) and V^1/2^.

The electrical properties of the studied samples are measured using the Keithley measurement source unit αModel 2400 SMU (Keithley Instruments, Cleveland, OH, USA). Our samples were measured in the nematic phase upon cooling. The samples were melted to 160 °C (isotropic liquid) and then allowed to cool to 115 °C for 30 min except for the nonmesomorphic sample (**I6_c_**) that was measured at room temperature (solid phase). The samples were provided with Ohmic contacts using silver paste (Resistivity < 0.04 Ω.cm). The current–voltage (*I–V*) response of the present synthesized analogs series, **I6_a–e_** films, are recorded by varying the applied voltage (V) from −10 V to 10 V with scan rates of 0.1 V and 0.5 V/s, as shown in Figure 8A,D. As the scan rate increased, the detected current increased, and the highest values are detected for the **I6_d_** film. The smallest values are detected for the **I6_d_** film. That indicates the existence of the terminal Cl group which greatly improves the flow of the charges in the film, and the opposite observation occurs with the existence of the F terminal group. It is evident that the observed behaviors are non-Ohmic (nonlinear). Thus, the change on the resistance of the materials occurred based on the current moving through it. Moreover, it has been recently noticed that the polymeric and organic systems are of Schottky diode behavior at low voltage. In the present investigation, the relation between log (I) and V^1/2^ shows two linear stages as illustrated in Figure 9, which implies that our prepared films follow the Schottky diode behavior. DC resistance and electrical conductance values of **I6_a–e_** films at a scan rate of 0.1 V/s and 0.5 V/s were measured and presented in Figure 8B–F. The samples have electrical resistances in the same order of magnitude as it has been shown in Figure 8B,E, even though the **I6_e_** film presents the highest resistance (743.3 GΩ) and **I6_d_** film shows the lowest resistance (106.5 GΩ). The resistance of **I6_e_** and **I6_d_** films are changed to 807.0 and 49.5 GΩ by increasing the scan rate to 0.5 V/s. Additionally, it is clearly shown in Figure 8C that the electrical conductance is decreased from 9.39 pS to 1.35 pS at scan rate 0.1 V/s by changing the terminal group from Cl to F. By increasing the scan rate to 0.5 V/s, the electric conductance changes to 20.22 pS for **I6_d_** and to 1.24 pS for **I6_e_**. These behaviors refer to the role of the terminal substituent in controlling the electric conductance, which depends mainly on the number and mobility of charge carriers [58,59]. The change in the density and mobility of charge carriers of the **I6_a–e_** samples is related to their anisotropy, molecular geometry, molecular biaxiality, molecular orientation, dipolar and π-π stacking interactions [43,50]. Hao et al. [60] reported robust dependency of the vertical charge carriers’ mobility on the π-π stacking gaps of the organic molecule/graphene heterojunction. In the same context, A.L. Briseno et al. [61] reported that the trapping of the charge carriers in the dipolar fields reduces the mobility of the films; Z. Ma et al. [62] conclude the increase of the charge mobility with the chain length which ascribed to the enhancement of the π-π interactions alongside the stacking directions and the expatriate stacking alongside the molecular plane short axis.

### 3.7. Optical Spectra and Energy Gap Calculation of **I6_a–e_** Films

**Figure 10:**
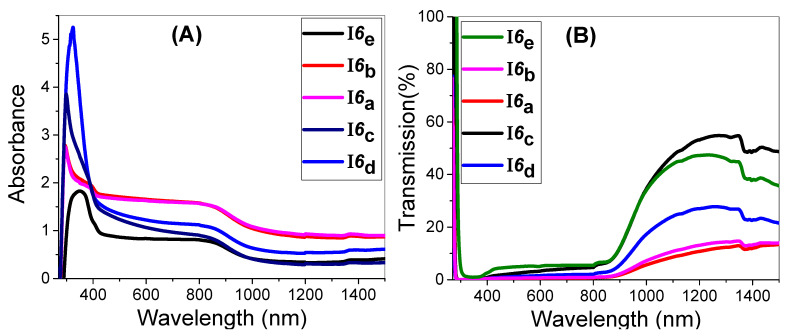
(**A**) Absorbance and (**B**) transmittance spectra for the present series **I6_a–e_** at 115 °C.

The optical absorbance and transmittance spectra of the prepared **I6_a–e_** films were measured using a Perkin Elmer spectrophotometer (*Lambda 900* UV-VIS-NIR) (PerkinElmer, Boston, MA, USA) within a wavelength range from 250 to 1500 nm. Figure 10A,B shows the dependence of the absorbance and transmittance of the films on the wavelength. From the optical spectra shown in Figure 10A, there is a very strong absorption band observed at 294 nm for **I6_a_**,**_b_**, at 298 nm for **I6_c_**, at 320 nm for **I6_d_**, and at 348 nm for **I6_e_**. The intensive UV absorption band is ascribed to the π–π* transition in the π-electronic system. The absorption in the UV and visible regions is higher than the absorption in the IR region. The samples showed almost plateau absorption in the visible light region, which decreased following the order: OCH_3_ > CH_3_ > Cl > H > F. Then, the absorption of the samples is decreased exponentially in the near IR range to reach almost a plateau absorption for λ > 1000 nm. From the transmission spectra shown in Figure 10B, the transmission of these samples is very low (< 10%) in the UV and visible regions. After that, the values of the transmission increase exponentially. The transmission of the samples is in order of H > F > Cl > CH_3_ > OCH_3._ The values of the maximum transmission in the IR region are in order of 55%, 47%, 28%, 15%, and 13%. This behavior confirms the role of the selected terminal substituents in enhancing the optical properties, which depend mainly on the morphology and chemical composition. Moreover, the observed red-shift in the absorption peak is mainly attributed to the size effect, in which small size reduces spin-orbit coupling and moderates the exciton positions [63]. This red-shift and high absorption in UV and visible regions is a desirable feature in energy-efficient solar cells [64].

**Figure 11:**
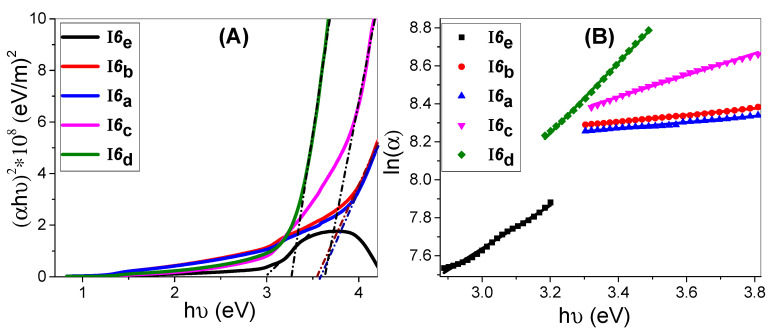
Calculation of (**A**) energy gap and (**B**) Urbach energy for the present series (**I6_a–e_**) at 115 °C.

The relationship between the absorption coefficient (*α*) and photon energy (*hv*) for the direct allowed transitions is defined by the optical absorption theorem (Equation (3)) [65]:

(*α*·*hv*)^2^ = *A* (*α*·*hv* − *Eg*) (3)

where *h* is the Planck’s constant (6.625 × 10^−34^ J/s), *A* is a constant, and *E_g_* is the optical bandgap. The values of direct *E_g_* for the prepared **I6_a–e_** films are obtained by extrapolating the linear portions of the plot of (*α*·*hν*)^2^ vs. *hν* to *α* = 0 as shown in Figure 11A. The linear parts observed in this figure indicate that the transitions are performed directly. The obtained values of *Eg* are displayed in Table 2. The smallest *Eg* value was determined to be 3.0 eV for **I6_e_** film and the highest value was determined to be 3.63 eV for **I6_c_** film. Due to the presence of the terminal fluorine atom, the observed decreasing in the bandgap of the nematogenic **I6e** film is attributable to the effect of the density of localized states. This result is compliant with the previously mentioned studies [66]. It is worthy to note the importance of the reduction of the bandgap values for the solar energy applications such as photoelectrochemical hydrogen generation solar cells.

The width of the exponential absorption edge is referred to by Urbach energy (EU) or the Urbach tail. The tails of the valence and conduction bands are attributed to the disorder in the material [64]. The EU’s exponential dependence can be measured using the following Equation (4) [67]:

*α* = *α*_o_*exp*(*hν*/*E_U_*) => *E_U_* = [*δ*·(*ln*(*α*))/*δ*·(*hν*)]^−1^ (4)

where *α_0_* is the band tailing parameter that can be obtained by (Equation (5)) [68];

*α*_o_ = [(4π/*c*)*·σ_o_/*χ·Δ*E*]^1/2^ (5)

where *c* is the speed of light, *σ_o_* is electrical conductivity at absolute zero, Δ*E* refers to the width of the tail of the localized state in the forbidden gap. Figure 11B shows the plot of ln(*α*) vs. *hν* for the prepared **I6_a–e_** films. The values of *E_U_* were obtained from the slopes of the linear fitting of these curves and presented in Table 2. The minimum value of *E_U_* was determined to be 160.9 mV for **I6_a_**, whereas the maximum value was determined to be 1858.2 mV for **I6_d_**.

## 4. Conclusions

New laterally methoxy-substituent photoactive liquid crystalline analogues series, named 4-hexyloxy phenyl- imino-4ʹ-(3-methoxyphenyl)-4ʹ’-alkoxybenzoates, were synthesized and mesomorphically characterized as well as optically evaluated. The prepared series included five analogues derivatives that differ from each other by the terminally compact polar substituent (OCH_3_, CH_3_, Cl, F) including the unsubstituted derivative (H). A laterally protruded OCH_3_ group is introduced into the central benzene ring and makes an angle of 120° with respect to the ester moiety. Molecular structures were elucidated by elemental analyses, FT-IR and NMR spectroscopy. Mesomorphic, optical, and electrical meaurements were conducted using DSC, POM, UV spectrophotometer, Keithley measurement-source unit, and UV/Vis/IR Perkin Elmer spectrophotometer. DSC and POM investigations indicated that all synthesized series are monomorphic, possessing the nematic mesophase, except the unsubstituted member (**I6_c_**) that is nonmesomorphic. Moreover, the polarizability anisotropy of the polar compact terminal and lateral groups influences the mesomeric characters of the whole molecular architecture. Furthermore, this nature of the terminal moieties influences the mesophase properties and induces phase transition phenomena when comparing to the unsubstituted derivative.

From the study of the electrical properties, the **I6_e_** film showed the highest resistance value of 743.3 GΩ and the **I6_d_** film showed the lowest resistance value of 106.5 GΩ at a scan rate of 0.1 V/s. The resistance of **I6_d_** film is decreased to be 49.5 GΩ by increasing the scan rate to 0.5 V/s. Moreover, the electrical conductance is decreased from 9.39 pS to 1.35 pS at scan rate 0.1 V/s by changing the terminal group from Cl to F. Moreover, there is a very strong absorption band centered at 294 nm for **I6_a_**,**_b_**, and 348 nm for **I6_e_**. The **I6_d_** showed very strong absorption relative to the other samples. Besides, the direct bandgap is tuned from 3.63 eV for **I6_c_** to 3.00 eV for **I6_e_** with band tails of 570.3 mV, and 1134.8 mV. Finally, the enhanced optical absorption and the reduced energy gap make the optimized samples suitable material for solar energy applications.

## Figures and Tables

**Table 1:** Mesomorphic transition temperatures (°C), enthalpy of transition Δ*H*, kJ/mole, mesophase range (Δ*T*) and the normalized entropy of transition Δ*S/R* for analogues series **I6_a–e_** upon heating cycle.

Comp.	X	*T*_Cr-N_	Δ*H*_Cr-N_	*T*_N-I_	Δ*H*_N-I_	Δ*T*_N_	Δ*S*_N-I_/R
**I6_a_**	OCH_3_	104.5	62.51	155.9	0.99	51.4	0.28
**I6_b_**	CH_3_	90.2	49.72	141.3	0.76	51.1	0.22
**I6_c_**	H	98.9	53.44	-	-	-	-
**I6_d_**	Cl	98.7	58.36	137.4	0.64	38.7	0.19
**I6_e_**	F	121.7	48.49	117.1 *	0.72 *	4.6 *	0.22

Cr-N = transition from solid to the nematic phase. N-I = transition from nematic to the isotropic liquid phase. * monotropic phase.

**Table 2:** Values of the energy gap (E_g_) and Urbach energy (E_U_) for analogs series **I6_a–e_**.

Comp.	Eg (eV)	Eu (meV)
**I6_a_**	3.58	160.9 ± 4.1
**I6_b_**	3.55	175.9 ± 2.3
**I6_c_**	3.63	570.3 ± 8.5
**I6_d_**	3.26	1858.2 ± 19.2
**I6_e_**	3.00	1134.8 ± 22.1

## Data Availability

The data presented in this study are available on request from corresponding authors.

## Supplementary Materials

The following are available online at https://www.mdpi.com/article/10.3390/ma14133718/s1, Figure S1: ^1^H-NMR spectra of compound I6a, Figure S2: ^13^C-NMR spectra of compound I6a, Figure S3: DSC thermograms of compounds I6e at a rate of 10 °C/min recorded from the second heating and cooling cycles.

## References

[1] Reyes C.G., Sharma A., Lagerwall J.P. (2016). Non-electronic gas sensors from electrospun mats of liquid crystal core fibres for detecting volatile organic compounds at room temperature. Liq. Cryst..

[2] Setia S., Sidiq S., De J., Pani I., Pal S.K. (2016). Applications of liquid crystals in biosensing and organic light-emitting devices: Future aspects. Liq. Cryst..

[3] Gupta R.K., Manjuladevi V., Karthik C., Choudhary K. (2016). Thin films of discotic liquid crystals and their applications. Liq. Cryst..

[4] Bhat S.G., Ramachandra G.S., Bhagavath P., Subrao M., Potukuchi D.M., Maddasani S. (2018). Self-assembled liquid crystalline materials with fatty acids. J. Therm. Anal. Calorim..

[5] Imrie C.T., Henderson P., Yeap G.-Y. (2009). Liquid crystal oligomers: Going beyond dimers. Liq. Cryst..

[6] Yeap G.Y., Lee H.C., Mahmood W.A.K., Imrie C.T., Takeuchi D., Osakada K. (2011). Synthesis, thermal and optical behaviour of non-symmetric liquid crystal dimers α-(4-benzylidene-substituted-aniline-4′-oxy)-ω-[pentyl-4-(4′-phenyl) benzoateoxy] hexane. Phase Transit..

[7] Yeap G.-Y., Osman F., Imrie C. (2015). Non-symmetric dimers: Effects of varying the mesogenic linking unit and terminal substituent. Liq. Cryst..

[8] Yeap G.Y., Hng T.C., Yeap S.Y., Gorecka E., Ito M.M., Ueno K., Okamoto M., Mahmood W.A.K., Imrie C.T. (2009). Why do non-symmetric dimers intercalate? The synthesis and characterisation of the α-(4-benzylidene-substituted-aniline-4′-oxy)-ω-(2-methylbutyl-4′-(4″-phenyl) benzoateoxy) alkanes. Liq. Cryst..

[9] Ahmed H.A., El-Atawy M.A. (2021). Synthesis, mesomorphic and geometrical approaches of new non-symmetrical system based on central naphthalene moiety. Liq. Cryst..

[10] El-Atawy M.A., Alhaddad O.A., Ahmed H.A. (2021). Experimental and geometrical structure characterizations of new synthesized laterally fluorinated nematogenic system. Liq. Cryst..

[11] Al-Zahrani S.A., Ahmed H.A., El-Atawy M.A., Abu Al-Ola K.A., Omar A.Z. (2021). Synthetic, Mesomorphic, and DFT Investigations of New Nematogenic Polar Naphthyl Benzoate Ester Derivatives. Materials.

[12] El-Atawy M.A., Naoum M.M., Al-Zahrani S.A., Ahmed H.A. (2021). New Nitro-Laterally Substituted Azomethine Derivatives; Synthesis, Mesomorphic and Computational Characterizations. Molecules.

[13] Al-Mutabagani L., Alshabanah L., Ahmed H., Alalawy H., Al Alwani M. (2021). Synthesis, Mesomorphic and Computational Characterizations of Nematogenic Schiff Base Derivatives in Pure and Mixed State. Molecules.

[14] Altowyan A., Ahmed H., Gomha S., Mostafa A. (2021). Optical and Thermal Investigations of New Schiff Base/Ester Systems in Pure and Mixed States. Polymers.

[15] Ahmed H., Hagar M., Alhaddad O. (2020). Mesomorphic and geometrical orientation study of the relative position of fluorine atom in some thermotropic liquid crystal systems. Liq. Cryst..

[16] Al-Mutabagani L., Alshabanah L., Ahmed H., El-Atawy M. (2021). Synthesis, Optical and DFT Characterizations of Laterally Fluorinated Phenyl Cinnamate Liquid Crystal Non-Symmetric System. Symmetry.

[17] Ionescu A., Godbert N., Aiello I., Crispini A., Ghedini M. (2012). Neutral and Cationic Cyclopalladated Nile Red Metallomesogens: Synthesis and Characterization In Memory of Dr. Teresa Pugliese. Mol. Cryst. Liq. Cryst..

[18] Al-Karawi A.J.M. (2017). From mesogens to metallomesogens. Synthesis, characterisation, liquid crystal and luminescent properties. Liq. Cryst..

[19] Ghedini M., Golemme A., Aiello I., Godbert N., Termine R., Crispini A., La Deda M., Lelj F., Amati M., Belviso S. (2011). Liaisons between photoconductivity and molecular frame in organometallic Pd(ii) and Pt(ii) complexes. J. Mater. Chem..

[20] Martinez-Felipe A., Lu Z., Henderson P.A., Picken S.J., Norder B., Imrie C.T., Ribes-Greus A. (2012). Synthesis and characterisation of side chain liquid crystal copolymers containing sulfonic acid groups. Polymer.

[21] Vicari L. (2016). Optical Applications of Liquid Crystals.

[22] Naoum M.M., Saad G.R., Nessim R.I., Abdel-Aziz T.A., Seliger H. (1997). Effect of molecular structure on the phase behaviour of some liquid crystalline compounds and their binary mixtures II. 4-Hexadecyloxyphenyl arylates and aryl 4-hexadecyloxy benzoates. Liq. Cryst..

[23] Saad G.R., Nessim R.I. (1999). Effect of molecular structure on the phase behaviour of some liquid crystalline compounds and their binary mixtures VI[1]. The effect of molecular length. Liq. Cryst..

[24] Naoum M.M., Mohammady S.Z., Ahmed H.A. (2010). Lateral protrusion and mesophase behaviour in pure and mixed states of model compounds of the type 4-(4′-substituted phenylazo)-2-(or 3-)methyl phenyl-4’-alkoxy benzoates. Liq. Cryst..

[25] Luckhurst G., Gray G.W. (1979). The Molecular Physics of Liquid Crystals.

[26] Ahmed H.A., Hagar M., El-Sayed T.H., Alnoman R.B. (2019). Schiff base/ester liquid crystals with different lateral substituents: Mesophase behaviour and DFT calculations. Liq. Cryst..

[27] Ahmed H.A., Khushaim M.S. (2020). Nematic Phase Induced from Symmetrical Supramolecular H-Bonded Systems Based on Flexible Acid Core. Crystals.

[28] Ahmed H., Khushaim M.S. (2020). Nematogenic Laterally Substituted Supramolecular H-Bonded Complexes Based on Flexible Core. Crystals.

[29] Al-Mutabagani L.A., Alshabanah L.A., Naoum M.M., Hagar M., Ahmed H.A. (2020). Experimental and Computational Approaches of Newly Polymorphic Supramolecular H-Bonded Liquid Crystal Complexes. Front. Chem..

[30] Dudley B. BP Statistical Review of World Energy. Proceedings of the World Petroleum Conference.

[31] Dong S., Zhang K., Xie B., Xiao J., Yip H.-L., Yan H., Huang F., Cao Y. (2019). High-Performance Large-Area Organic Solar Cells Enabled by Sequential Bilayer Processing via Nonhalogenated Solvents. Adv. Energy Mater..

[32] Wang G., Adil M.A., Zhang J., Wei Z. (2019). Large-Area Organic Solar Cells: Material Requirements, Modular Designs, and Printing Methods. Adv. Mater..

[33] Fan B., Zhang D., Li M., Zhong W., Zeng Z., Ying L., Huang F., Cao Y. (2019). Achieving over 16% efficiency for single-junction organic solar cells. Sci. China Ser. B Chem..

[34] Ahmed A.M., Abdalla E.M., Shaban M. (2020). Simple and Low-Cost Synthesis of Ba-Doped CuO Thin Films for Highly Efficient Solar Generation of Hydrogen. J. Phys. Chem. C.

[35] Shaban M., El Sayed A.M. (2020). Influence of the spin deposition parameters and La/Sn double doping on the structural, optical, and photoelectrocatalytic properties of CoCo_2_O_4_ photoelectrodes. Sol. Energy Mater. Sol. Cells.

[36] Shaban M., Hamd A., Amin R.R., Abukhadra M.R., Khalek A.A., Khan A.A.P., Asiri A.M. (2020). Preparation and characterization of MCM-48/nickel oxide composite as an efficient and reusable catalyst for the assessment of photocatalytic activity. Environ. Sci. Pollut. Res..

[37] Helmy A., Rabia M., Shaban M., Ashraf A.M., Ahmed S., Ahmed A.M. (2020). Graphite/rolled graphene oxide/carbon nanotube photoelectrode for water splitting of exhaust car solution. Int. J. Energy Res..

[38] Mohamed F., Rabia M., Shaban M. (2020). Synthesis and characterization of biogenic iron oxides of different nanomorphologies from pomegranate peels for efficient solar hydrogen production. J. Mater. Res. Technol..

[39] Gomha S.M., Muhammad Z.A., Abdel-Aziz H.M., Matar I.K., El-Sayed A.A. (2020). Green synthesis, molecular docking and anticancer activity of novel 1,4-dihydropyridine-3,5-Dicarbohydrazones under grind-stone chemistry. Green Chem. Lett. Rev..

[40] Gomha S.M., Edrees M.M., Muhammad Z.A., Kheder N.A., Melha S.A., Saad A.M. (2020). Synthesis, Characterization, and Antimicrobial Evaluation of Some New 1,4-Dihydropyridines-1,2,4-Triazole Hybrid Compounds. Polycycl. Aromat. Compd..

[41] Gomha S.M., Riyadh S.M. (2011). Synthesis under Microwave Irradiation of [1,2,4]Triazolo[3,4-b][1,3,4]thiadiazoles and Other Diazoles Bearing Indole Moieties and Their Antimicrobial Evaluation. Molecules.

[42] Abu-Melha S., Edrees M.M., Riyadh S.M., Abdelaziz M.R., ElFiky A.A., Gomha S.M. (2020). Clean Grinding Technique: A Facile Synthesis and In Silico Antiviral Activity of Hydrazones, Pyrazoles, and Pyrazines Bearing Thiazole Moiety against SARS-CoV-2 Main Protease (M^pro^). Molecules.

[43] Sayed A.R., Gomha S.M., A Taher E., A Muhammad Z., El-Seedi H.R., Gaber H.M., Ahmed M.M. (2020). One-Pot Synthesis of Novel Thiazoles as Potential Anti-Cancer Agents. Drug Des. Dev. Ther..

[44] Gomha S.M., Eldebss T.M.A., Badrey M.G., Abdulla M.M., Mayhoub A.S. (2015). Novel 4-Heteroaryl-Antipyrines as DPP-IV Inhibitors. Chem. Biol. Drug Des..

[45] Gomha S.M., Kheder N.A., Abdelaziz M.R., Mabkhot Y., Alhajoj A.M. (2017). A facile synthesis and anticancer activity of some novel thiazoles carrying 1,3,4-thiadiazole moiety. Chem. Cent. J..

[46] Gomha S.M., Kheder N.A., Abdelhamid A.O., Mabkhot Y.N. (2016). One Pot Single Step Synthesis and Biological Evaluation of Some Novel Bis(1,3,4-thiadiazole) Derivatives as Potential Cytotoxic Agents. Molecules.

[47] Attard G., Imrie C., Karasz F. (1992). Low molar mass liquidcrystalline glasses: Preparation and properties of the. alpha.-(4-cyanobiphenyl-4′-oxy)-. omega.-(1-pyrenimin ebenzylidene-4′-oxy) alkanes. Chem. Mater..

[48] Attard G.S., Imrie C.T. (1992). Liquid-crystalline and glass-forming dimers derived from 1-aminopyrene. Liq. Cryst..

[49] Abberley J.P., Jansze S.M., Walker R., Paterson D.A., Henderson P.A., Marcelis A.T.M., Storey J., Imrie C.T. (2017). Structure–property relationships in twist-bend nematogens: The influence of terminal groups. Liq. Cryst..

[50] Blatch A.E., Fletcher I.D., Luckhurst G.R. (1995). The intercalated smectic-A phase—the liquid-crystal properties of the α-(4-cyanobiphenyl-4ʹ-yloxy)-ω-(4-alkyloxycinnamoate) alkanes. Liq. Cryst..

[51] Veen J.V. (1975). The influence of terminal substituents upon the nematic-isotropic transition temperature. Le Journal de Physique Colloques. J. Phys. Colloq..

[52] Naoum M.M., Nessim R.I., Saad G.R., Aziz T.A.A. (1998). Effect of molecular structure on the phase behaviour of some liquid crystalline compounds and their binary mixtures IV. Dependence of Tc on the anisotropy of the aryl-X of polarizability bond. Liq. Cryst..

[53] Nafee S.S., Hagar M., Ahmed H.A., El-Shishtawy R.M., Raffah B.M. (2019). The Synthesis of New Thermal Stable Schiff Base/Ester Liquid Crystals: A Computational, Mesomorphic, and Optical Study. Molecules.

[54] Bakhtiari G., Moradi S., Soltanali S. (2014). A novel method for the synthesis of coumarin laser dyes derived from 3-(1H-benzoimidazol-2-yl) coumarin-2-one under microwave irradiation. Arab. J. Chem..

[55] Hrozhyk U.A., Serak S.V., Tabiryan N.V., Hoke L., Steeves D.M., Kimball B., Kedziora G. (2008). Systematic Study of Absorption Spectra of Donor–Acceptor Azobenzene Mesogenic Structures. Mol. Cryst. Liq. Cryst..

[56] Saint-Jalm S., Miniewicz A., Karpinski P., Jarek-Mikulska U., Galewski Z. (2012). Photo-induced birefringence in a nematic liquid crystal mixture doped with light-switchable mesogenic azobenzene derivatives. J. Mol. Liq..

[57] Naoum M.M., Fahmi A.A., Saad G.R., Ali M.H. (2017). Polarity and steric effect of di-lateral substitution on the mesophase behaviour of some azo/ester compounds. Liq. Cryst..

[58] Rathi S., Chauhan G., Gupta S.K., Srivastava R., Singh A. (2017). Analysis of blockade in charge transport across polymeric heterojunctions as a function of thermal annealing: A different perspective. J. Electron. Mater..

[59] Kumar M., Kumar S., Upadhyaya A., Yadav A., Gupta S.K., Singh A. (2018). Study of charge transport in composite blend of P3HT and PCBM. AIP Conference Proceedings.

[60] Hao W., Wang Y., Zhao H., Zhu J., Li S. (2020). Strong dependence of the vertical charge carrier mobility on the π–π stacking distance in molecule/graphene heterojunctions. Phys. Chem. Chem. Phys..

[61] Briseno A.L., Miao Q., Ling M.-M., Reese C., Meng H., Bao Z., Wudl F. (2006). Hexathiapentacene: Structure, Molecular Packing, and Thin-Film Transistors. J. Am. Chem. Soc..

[62] Ma Z., Geng H., Wang N., Shuai Z. (2016). Influence of alkyl side-chain length on the carrier mobility in organic semiconductors: Herringbone vs. pi–pi stacking. J. Mater. Chem. C.

[63] Shaban M., El Sayed A. (2016). Effects of lanthanum and sodium on the structural, optical and hydrophilic properties of sol–gel derived ZnO films: A comparative study. Mater. Sci. Semicond. Process..

[64] Liu S., Kan Z., Thomas S., Cruciani F., Brédas J.-L., Beaujuge P.M. (2016). Thieno[3,4-c]pyrrole-4,6-dione-3,4-difluorothiophene Polymer Acceptors for Efficient All-Polymer Bulk Heterojunction Solar Cells. Angew. Chem. Int. Ed..

[65] Shaban M., El Sayed A. (2015). Influences of lead and magnesium co-doping on the nanostructural, optical properties and wettability of spin coated zinc oxide films. Mater. Sci. Semicond. Process..

[66] Mullekom H.A.M. (2000). The Chemistry of High and Low Band Gap π-Conjugated Polymers.

[67] El Sayed A., Shaban M. (2015). Structural, optical and photocatalytic properties of Fe and (Co, Fe) co-doped copper oxide spin coated films. Spectrochim. Acta Part A Mol. Biomol. Spectrosc..

[68] Sharma S., Vyas S., Periasamy C., Chakrabarti P. (2014). Structural and optical characterization of ZnO thin films for optoelectronic device applications by αsputtering technique. Superlattice Microst..

