# Supplementary material for: Synthesis, Optical Characterizations and Solar Energy Applications of New Schiff Base Materials
Source: Materials (Basel). 2021 Jul 2;14(13):3718. doi: 10.3390/ma14133718 (PMC8269905; doi:10.3390/ma14133718)
Supplement: Supplementary file 1 [file materials-14-03718-s001.zip › materials-1269485-supplementary.pdf]

Supplementary data

Synthesis, Optical Characterizations and Solar Energy Applications of New Schiff Base Materials

Sobhi M. Gomha[1]*, Hoda A. Ahmed [2,3*], Mohamed Shaban [4] , Tariq Z. Abolibda[1], Muna S. Khushaim[5,6] and Khalid A. Alharbi[1]

1. *Chemistry Department, Faculty of Science, Islamic University in Almadinah Almonawara, Almadinah Almonawara, 42351, Saudi Arabia, s.m.gomha@gmail.com (SG); t.z.a@iu.edu.sa (TA)*
2. *Department of Chemistry, Faculty of Science, Cairo University, Cairo 12613, Egypt.*
3. *Chemistry Department, College of Sciences, Yanbu, Taibah University, Yanbu 30799, Saudi Arabia.*
4. *Department of Physics, Faculty of Science, Islamic University in Almadinah Almonawara, Almadinah, 42351, Saudi Arabia, mssfadel@aucegypt.edu*
5. *Department of Physics, Faculty of Science, Taibah University, P. O. Box: 30002, P. Code 41447, Al-Madina, Saudi Arabia. (M.S.Kh) mkhushaim@taibahu.edu.sa*
6. *Nanotechonolgy Center, Taibah University, P. Box: 30002, P. Code 41447, Al-Madina, Saudi Arabia*

* *Correspondence:* sm.gomha@iu.edu.sa *(S.M. G); ahoda@sci.cu.edu.eg (H.A. A.)*

1. *Materials*

4-Hexyloxyaniline, 4-hydroxy-2-methoxybenzaldehyde, benzoic acid, 4-methylbenzoic acid, 4-methoxybenzoic acid, 4-fluorobenzoic acid and 4-chlorobenzoic acid were purchased from Sigma Aldrich (Germany). dichloromethane, *N,N'*-dicyclohexylcarbodiimide (DCC), ethanol and 4-dimethylaminopyridine (DMAP) were purchased from Aldrich (Wisconsin, USA).

2. *Synthesis of* 4-(((4-(hexyloxy)phenyl)imino)methyl)-3-methoxyphenol, *3*

A mixture of 4–hydroxy-2-methoxybenzaldehyde (1.52g, 10 mmol) and 4-(hexyloxy)aniline (1.93g, 10 mmol) in ethanol (20 mL) were refluxed for two hours (monitored by TLC). The mixture was cooled to room temperature and filtered. The obtained solid was washed with cold ethanol and recrystallized twice from hot ethanol to give pure imine compound **3** as indicated by TLC analysis. The melting points and IR data determined of the prepared imine **3:** Yield: 90.1%; mp 101.1 $^{\circ}$C, FTIR (ύ,

cm^{-1}): 3400 (OH), 2928, 2832 (CH_2 stretching), 1733 (C=O), 1609 (C=N), 1566 (C=C), 1466 (C–O $_{Asym}$), 1251 (C-O $_{Sym}$). ^{1}H-NMR (400 MHz, $CDCl_3$): δ/ppm: 0.91 (t, 3H, $\underline{CH_3}(CH_2)_3CH_2CH_2O$-), 1.28-1.43 (m, 6H, $CH_3\underline{(CH_2)_3}CH_2CH_2O$-), 1.71-1.76 (q, 2H, $CH_3(CH_2)_3\underline{CH_2}CH_2O$-), 3.72 (s, 1H, OH), 3.93 (s, 3H, O$\underline{CH_3}$), 4.08 (t, 2H, $CH_3(CH_2)_3CH_2\underline{CH_2}O$-), 6.79(d, 2H, Ar–H), 6.79 (d, 2H, Ar–H), 6.89 (d, 2H, Ar–H), 7.01 (d, 2H, Ar–H), 7.24 (s, 1H, Ar–H), 7.32 (d, 2H, Ar–H), 9.76 (s, 1H, CH=N). Anal. Calcd. for $C_{20}H_{25}NO_3$ (327.42): C, 73.37; H, 7.70; N, 4.28. Found: C, 73.21; H, 7.59; N, 4.13%.

3. *General procedure for synthesis of 4-(((4-(Hexyloxy)phenyl)imino)methyl)-3-methoxyphenyl 4-substituted benzoates, I6a-e*

A mixture of imine compound **3** (1.93g, 10 mmol) and the appropriate 4-substituted benzoic acid derivatives (10 mmol for each) in dry methylene chloride (25 mL) containing *N*, *N′*–dicyclohexylcarbodiimide (DCC, 10 mmol) and few crystals of 4–dimethylaminopyridine (DMAP), as catalyst, were left to react for 72 hours at room temperature with continuous stirring. The solid separated was then filtered off and the solution evaporated. The solid residue obtained was recrystallized from ethanol to give TLC pure products. The purity of the prepared samples was checked with thin-layer chromatography (TLC) using TLC sheets coated with silica gel (E Merck), and CH_2Cl_2/CH_3OH (9:1) as eluent, whereby only one spot was detected by a UV-lamp.

Infrared spectra (IR), ^{1}H-NMR, and elemental analyses for investigated compounds were consistent with the structures assigned. ^{1}H-NMR data showed the expected integrated aliphatic to aromatic proton ratios in all compounds investigated. The physical data of products **I6a-e** are listed below:

4-(((4-(Hexyloxy)phenyl)imino)methyl)-3-methoxyphenyl 4-methoxybenzoate **I*6*$_a$:**
Yield: 90.7%; mp 104.5 °C, FTIR (ύ, cm^{-1}): 2923, 2847 (CH_2 stretching), 1731 (C=O), 1608 (C=N), 1586 (C=C), 1466 (C–O $_{Asym}$), 1251 (C-O $_{Sym}$). ^{1}H-NMR (400 MHz, $CDCl_3$): δ/ppm: 0.91 (t, 3H, $\underline{CH_3}(CH_2)_3CH_2CH_2O$-), 1.23-1.34 (m, 6H, $CH_3\underline{(CH_2)_3}CH_2CH_2O$-), 1.72-1.74 (q, 2H, $CH_3(CH_2)_3\underline{CH_2}CH_2O$-), 3.83 (s, 3H, O$\underline{CH_3}$), 3.89 (s, 3H, O$\underline{CH_3}$), 3.96-3.99 (t, 2H, $CH_3(CH_2)_3CH_2\underline{CH_2}O$-), 6.91(d, 2H, Ar–H), 6.97 (d, 1H, Ar–H), 7.21(d, 2H, Ar–H), 7.23 (d, 2H, Ar–H), 7.69 (s, 1H,

Ar–H), 8.16-8.20 (m, 3H, Ar–H), 8.45 (s, 1H, CH=N). ^{13}C-NMR (400 MHz, $CDCl_3$): *δ*/ppm: 14.09 (CH_3), 18.49, 22.68, 24.75, 25.54 (CH_2), 55.81, 58.22 (OCH_3), 68.37 (CH_2-O), 106.65, 110.56, 113.91, 115.04, 122.24, 122.88, 123.23, 129.16, 132.53, 135.43, 139.87, 142.67 (Ar-C), 152.06 (C=N), 157.44, 158.09 (Ar-C), 163.99 (C=O). Anal. Calcd. for $C_{28}H_{31}NO_5$ (461.55): C, 72.86; H, 6.77; N, 3.03. Found: C, 72.64; H, 6.59; N, 3.00%.

4-(((4-(Hexyloxy)phenyl)imino)methyl)-3-methoxyphenyl 4-methylbenzoate **I6$_b$**: Yield: 88.0%; mp 90.2 °C, FTIR (ύ, cm^{-1}): 2922, 2863 (CH_2 stretching), 1728 (C=O), 1609 (C=N), 1587 (C=C), 1460 (C-O $_{Asym}$), 1269 (C-O $_{Sym}$). ^{1}H-NMR (400 MHz, $CDCl_3$): *δ*/ppm: 0.94 (t, 3H, $CH_3(CH_2)_3CH_2CH_2O$-), 1.20-1.33 (m, 6H, $CH_3(CH_2)_3CH_2CH_2O$-), 1.71-1.73 (q, 2H, $CH_3(CH_2)_3CH_2CH_2O$-), 2.36 (s, 3H, Ar-CH_3), 3.89 (s, 3H, OCH_3), 3.91-4.01 (t, 2H, $CH_3(CH_2)_3CH_2CH_2O$-), 6.90 (d, 2H, Ar–H), 7.16 (d, 1H, Ar–H), 7.20 (d, 2H, Ar–H), 7.41 (d, 2H, Ar–H), 7.72 (s, 1H, Ar–H), 8.09 (d, 1H, Ar–H), 8.17 (d, 2H, Ar–H), 8.44 (s, 1H, CH=N). Anal. Calcd. for $C_{28}H_{31}NO_4$ (445.55): C, 75.48; H, 7.01; N, 3.14. Found: C, 75.30; H, 6.88; N, 3.05%.

4-(((4-(Hexyloxy)phenyl)imino)methyl)-3-methoxyphenyl benzoate **I6$_c$**:
Yield: 90.5%; mp 98.9 °C, FTIR (ύ, cm^{-1}): 2925, 2867 (CH_2 stretching), 1722 (C=O), 1612 (C=N), 1584 (C=C), 1461 (C–O $_{Asym}$), 1259 (C–O $_{Sym}$). ^{1}H-NMR (400 MHz, $CDCl_3$): *δ*/ppm: 0.93 (t, 3H, *J* = 8 Hz, $CH_3(CH_2)_3CH_2CH_2O$-), 1.36-1.38 (m, 6H, $CH_3(CH_2)_3CH_2CH_2O$-), 1.78-1.85 (q, 2H, *J* = 4 Hz, $CH_3(CH_2)_3CH_2CH_2O$-), 3.93(s, 3H, OCH_3), 3.98-4.01 (t, 2H, *J* = 4 Hz, $CH_3(CH_2)_3CH_2CH_2O$-), 6.94 (d, 2H, Ar–H), 7.25 (d, 1H, Ar–H), 7.41 (d, 2H, Ar–H),7.53 (t, 2H, Ar–H), 7.66 (t, 1H, Ar–H), 7.76 (s, 1H, Ar–H), 8.21-8.25 (m, 3H, Ar–H), 8.47 (s, 1H, CH=N). Anal. Calcd. for $C_{27}H_{29}NO_4$ (431.52): C, 75.15; H, 6.77; N, 3.25. Found: C, 75.04; H, 6.59; N, 3.14%.

4-(((4-(Hexyloxy)phenyl)imino)methyl)-3-methoxyphenyl 4-chlorobenzoate **I6$_d$**:
Yield: 89.8%; mp 98.7 °C, FTIR (ύ, cm^{-1}): 2918, 2850 (CH_2 stretching), 1723 (C=O), 1606 (C=N), 1579 (C=C), 1467 (C-O $_{Asym}$), 1262 (C-O $_{Sym}$). ^{1}H-NMR (400 MHz, $CDCl_3$): *δ*/ppm: 0.93 (t, 3H, *J* = 8 Hz, $CH_3(CH_2)_3CH_2CH_2O$-), 1.23-1.37 (m, 6H, $CH_3(CH_2)_3CH_2CH_2O$-), 1.77-1.84 (q, 2H, *J* = 8 Hz, $CH_3(CH_2)_3CH_2CH_2O$-), 3.90 (s, 3H, OCH_3), 3.98-4.01 (t, 2H, *J* = 8 Hz, $CH_3(CH_2)_3CH_2CH_2O$-), 6.93 (d, 2H, Ar–H),

6.97 (d, 1H, Ar–H), 7.24 (d, 2H, Ar–H), 7.52 (d, 2H, Ar–H), 7.74 (s, 1H, Ar–H), 8.14-8.18 (m, 3H, Ar–H), 8.47 (s, 1H, CH=N). Anal. Calcd. for $C_{27}H_{28}ClNO_4$ (465.97): C, 69.59; H, 6.06; N, 3.01. Found: C, 69.73; H, 6.01; N, 2.94%.

4-(((4-(Hexyloxy)phenyl)imino)methyl)-3-methoxyphenyl 4-fluorobenzoate **I6e**:
Yield: 92.3%; mp 121.7 °C, FTIR (ύ, cm^{-1}): 2924, 2874 (CH_2 stretching), 1725 (C=O), 1606 (C=N), 1587 (C=C), 1460 ($C\text{-}O_{Asym}$), 1255 ($C\text{-}O_{Sym}$). ^{1}H-NMR (400 MHz, $CDCl_3$): δ/ppm: 0.94 (t, 3H, $\underline{CH_3}(CH_2)_3CH_2CH_2O$-), 1.38-1.52 (m, 6H, $CH_3\underline{(CH_2)_3}CH_2CH_2O$-), 1.79-1.85 (q, 2H, $CH_3(CH_2)_3\underline{CH_2}CH_2O$-), 3.92 (s, 3H, $O\underline{CH_3}$), 3.99-4.03 (t, 2H, $CH_3(CH_2)_3CH_2\underline{CH_2}O$-), 6.93 (d, 2H, Ar–H), 7.20-7.29 (m, 5H, Ar–H), 7.78 (s, 1H, Ar–H), 8.25-8.29 (m, 3H, Ar–H), 8.49 (s, 1H, CH=N). Anal. Calcd. for $C_{27}H_{28}FNO_4$ (449.51): C, 72.14; H, 6.28; N, 3.12. Found: C, 72.01; H, 6.37; N, 3.04%.
Schiff base derivative **3**, prepared by the reaction of aniline derivative **1** with substituted benzaldehyde derivative **2** in ethanol under reflux, was used for preparation of 4-(((4-(hexyloxy)phenyl)imino)methyl)-3-methoxyphenyl 4-substituted benzoates **I6a-e** *via* its reaction with the appropriate 4-substituted benzoic acid **4a-e** in methylene chloride containing *N*, *N′*–dicyclohexylcarbodiimide (DCC) and catalytic amounts of 4-dimethylaminopyridine (DMAP) at room temperature.

For example, the ^{1}H-NMR (400 MHz, $CDCl_3$) spectra of compound ***I6a*** exhibited four singlet signals at δ = 3.83, 3.89, 7.69, 8.45 ppm assignable for 2 OCH_3, Ar–H and azomethine proton, CH=N, respectively in addition to the characteristic signals for n-hexyl protons and ten aromatic protons (**Figure S1**).

^{13}C-NMR (400 MHz, $CDCl_3$) for compound ***I6a*** showed the characteristic signals for 2OCH_3 groups at δ 55.81, 58.22 ppm, respectively, n-hexyl group at δ = 14.09 (CH_3), 18.49, 22.68, 24.75, 25.54 (CH_2), 68.37 (CH_2-O) ppm, 14 aromatic carbons at δ = 106.65, 110.56, 113.91, 115.04, 122.24, 122.88, 123.23, 129.16, 132.53, 135.43, 139.87, 142.67 157.44, 158.09 ppm, in addition to signals at δ = 152.06, 163.99 ppm assignable to C=N and C=O carbons, respectively (**Figure S2**).

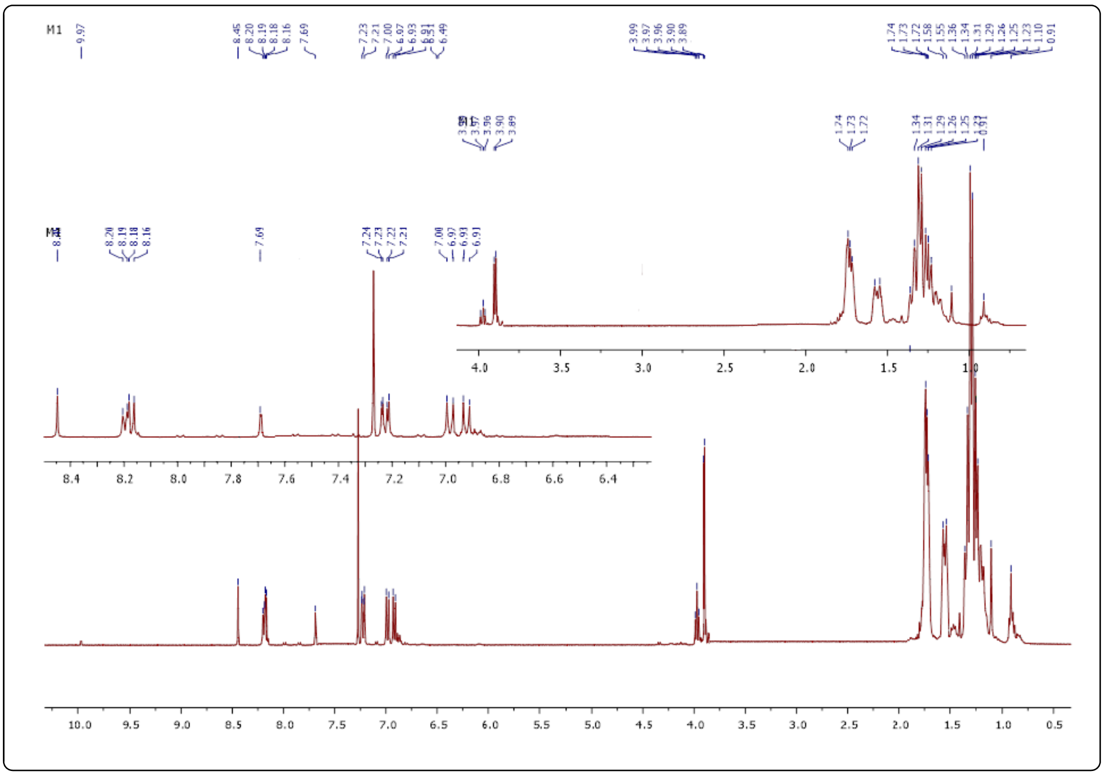

Figure S1. ^{1}H-NMR spectra of compound **I6$_{a}$**

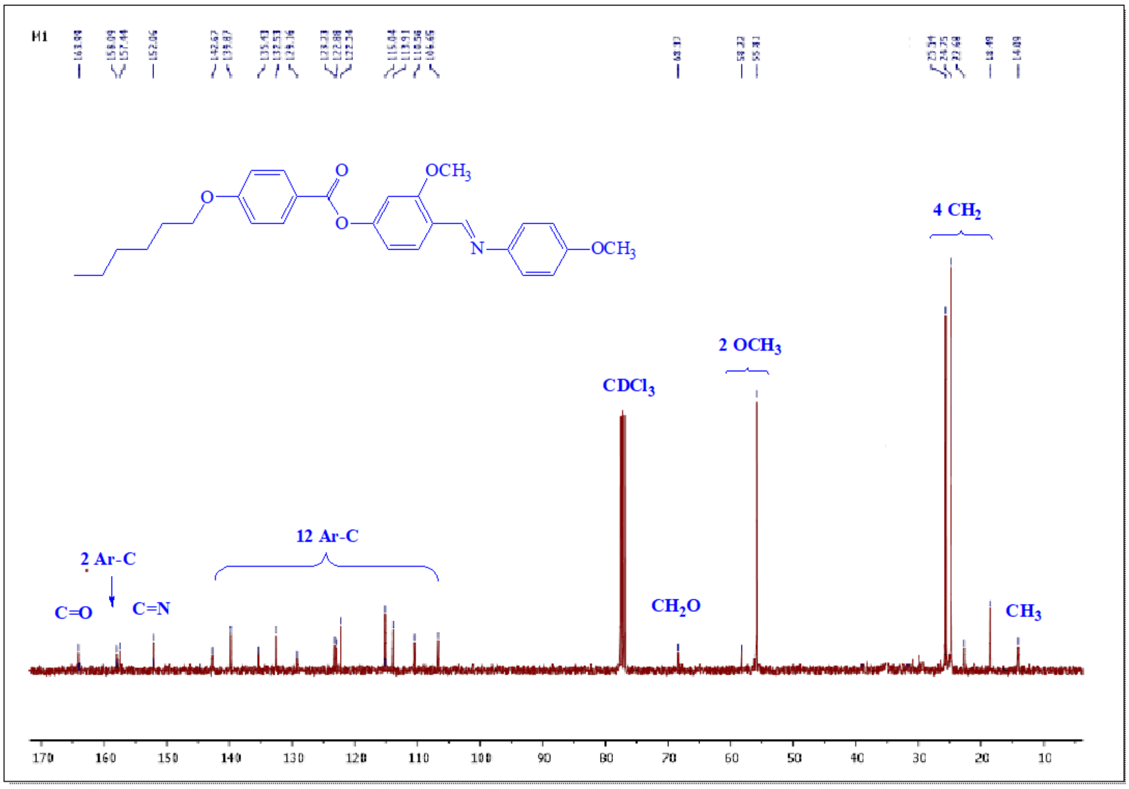

Figure S2. ^{13}C-NMR spectra of compound **I6$_{a}$**

4. Characterizations and instrumentation

Purity of all prepared compounds were checked with thin-layer chromatography using TLC-sheets coated with silica gel (E. Merck), whereby single spots were detected by a UV-lamp.

Perkin-Elmer B25 (Perkin-Elmer, Inc., Shelton, CT USA) spectrophotometer was used for infrared spectra measurements. Varian EM 350L 500 MHz spectrometer (Oxford, UK) was used for recording ^{1}HNMR spectra using tetramethyl silane as internal standard in $CDCl_3$; the chemical shift values recorded as δ (in ppm units). Thermo Scientific Flash 2000 CHS/O Elemental Analyzer, Milan, Italy was used for Elemental analyses.

The spectrophotometer (UV-1800 SHIMADZU, Japan) was used for UV-Vis measurements in a wavelength ranging from 200–800 nm at 25 ºC.

TA Instruments Co. (Q20 Differential Scanning Calorimeter, DSC; USA) was used for recording phase transitions. DSC calibration was carried out using lead and indium to calibrate the melting temperatures and enthalpies. Samples of 2–3 mg were used in aluminum pans for DSC investigation. The heating rate was 10°C/min in nitrogen gas as an inert atmosphere (30 ml/min). All transitions temperatures were measured form the second heating scan.

Transition temperatures for the prepared compounds were checked and phases identified by Polarized optical microscope (POM, Wild, Germany) attached with Mettler FP82HT hot stage.

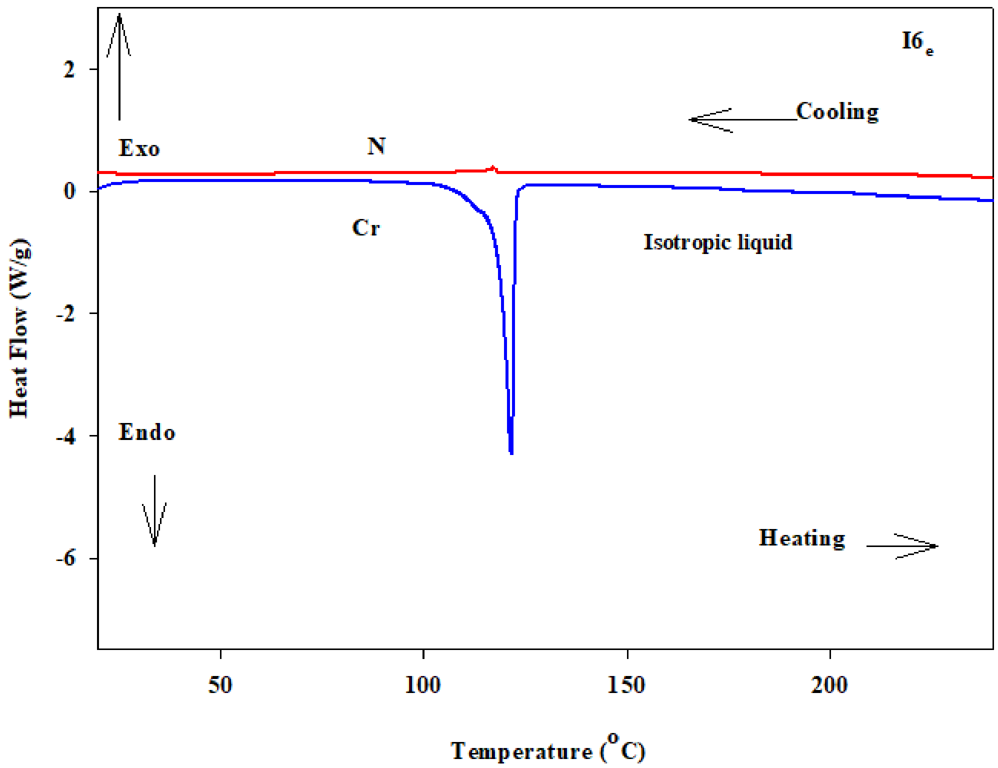

Figure S3: DSC thermograms of compounds **I6$_e$** at a rate of ±10°C/min recorded from the second heating and cooling cycles**.**
